# Supplementary material for: Doubly protected ester prodrug of 5-aminolevulinic acid for enhanced cell uptake and photoinactivation
Source: Photochem Photobiol Sci. 2026 Jul 21;25(9):1687–99. doi: 10.1007/s43630-026-00944-7 (PMC13562132; doi:10.1007/s43630-026-00944-7)
Supplement: Supplementary file 1 [file 43630_2026_944_MOESM1_ESM.pdf]

Electronic Supplementary Information for:

Doubly protected ester prodrug of 5-aminolevulinic acid for enhanced cell uptake and photoinactivation

Hailey S. Sanders, Karolina A. Rooney, Marissa A. Panethiere, Kirk M. Atkinson, and Bradley D. Smith*

Department of Chemistry and Biochemistry,
University of Notre Dame,
Notre Dame, Indiana
46556, USA
*E-mail: smith.115@nd.edu

Compound Spectra

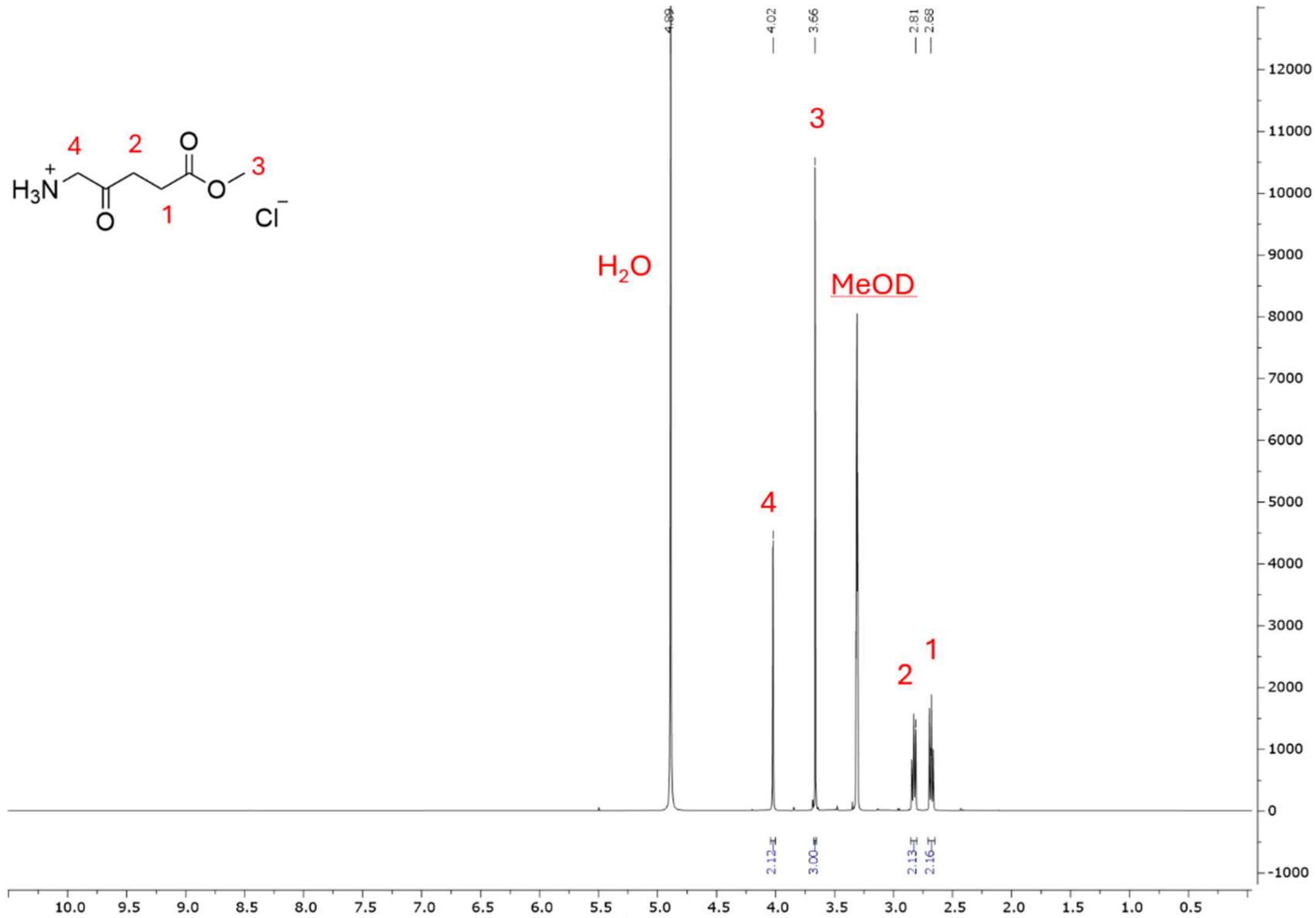

^{1}HNMR (400 MHz, MeOD) of **MAL**

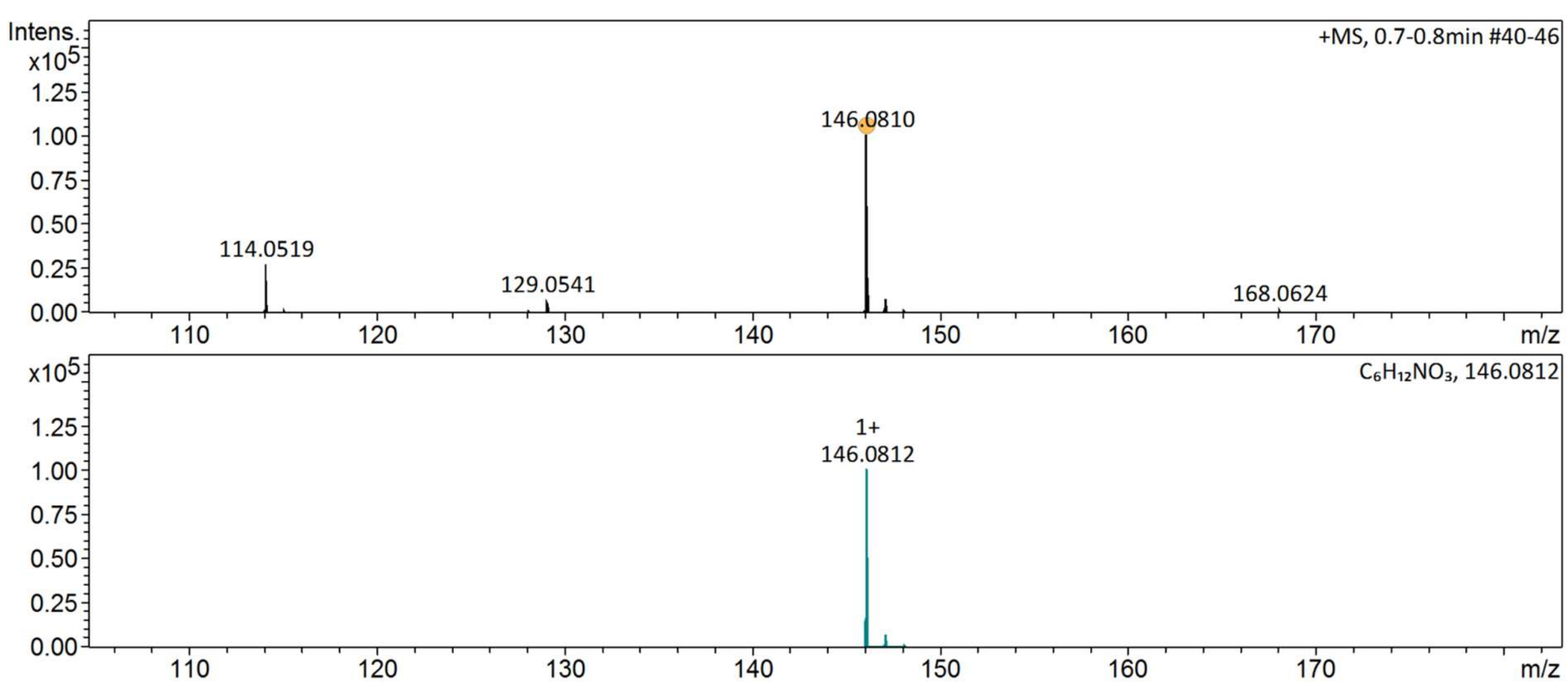

HRMS (SI-TOF) spectrum of **MAL**

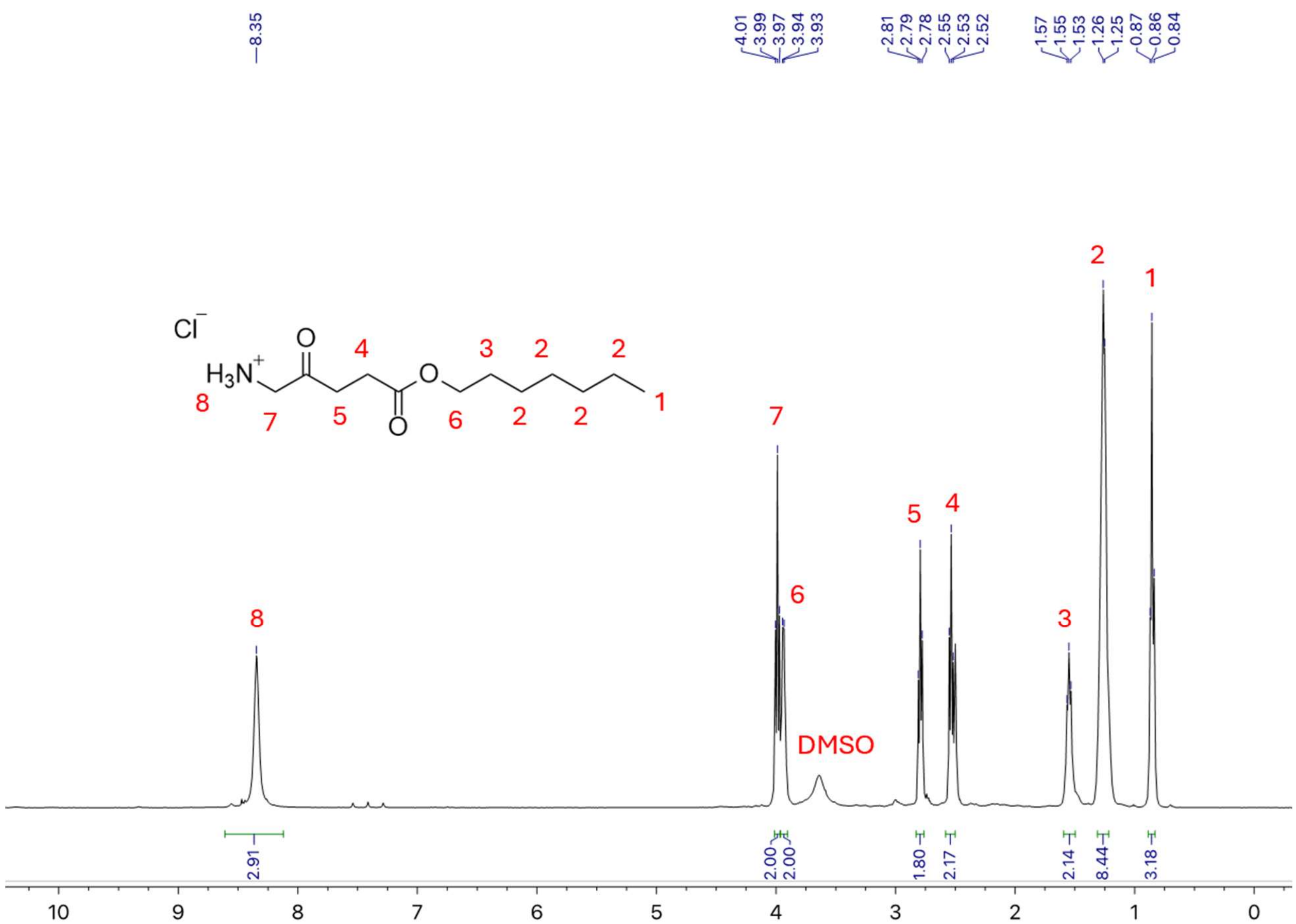

^{1}H NMR spectrum (400 MHz, DMSO-d_6) of **HAL**

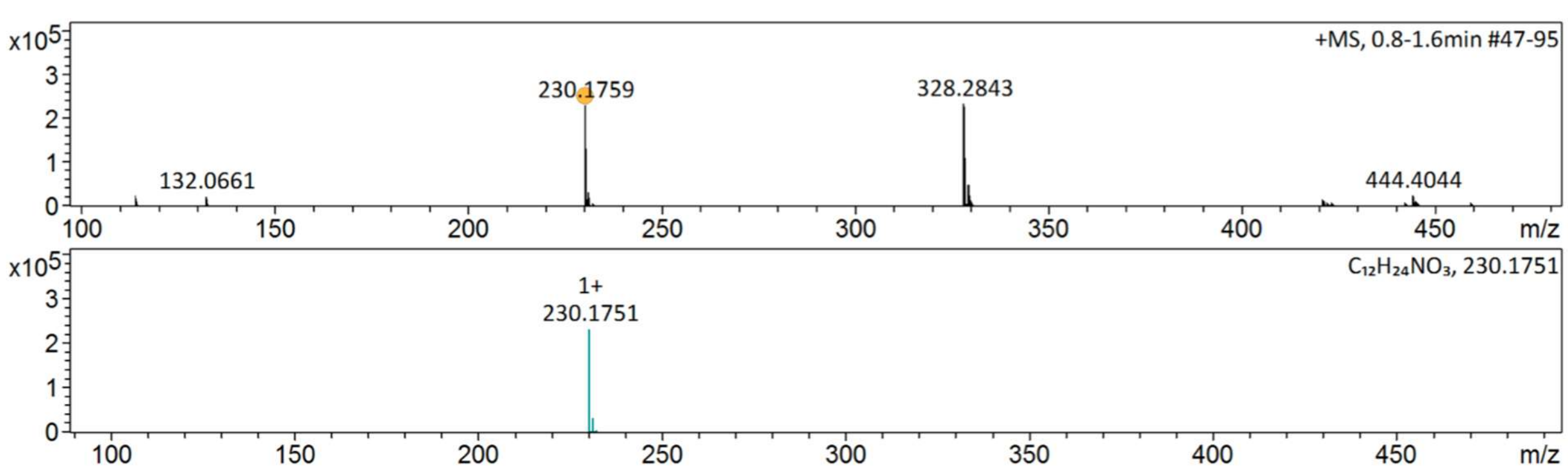

HRMS (SI-TOF) spectrum of **HAL**

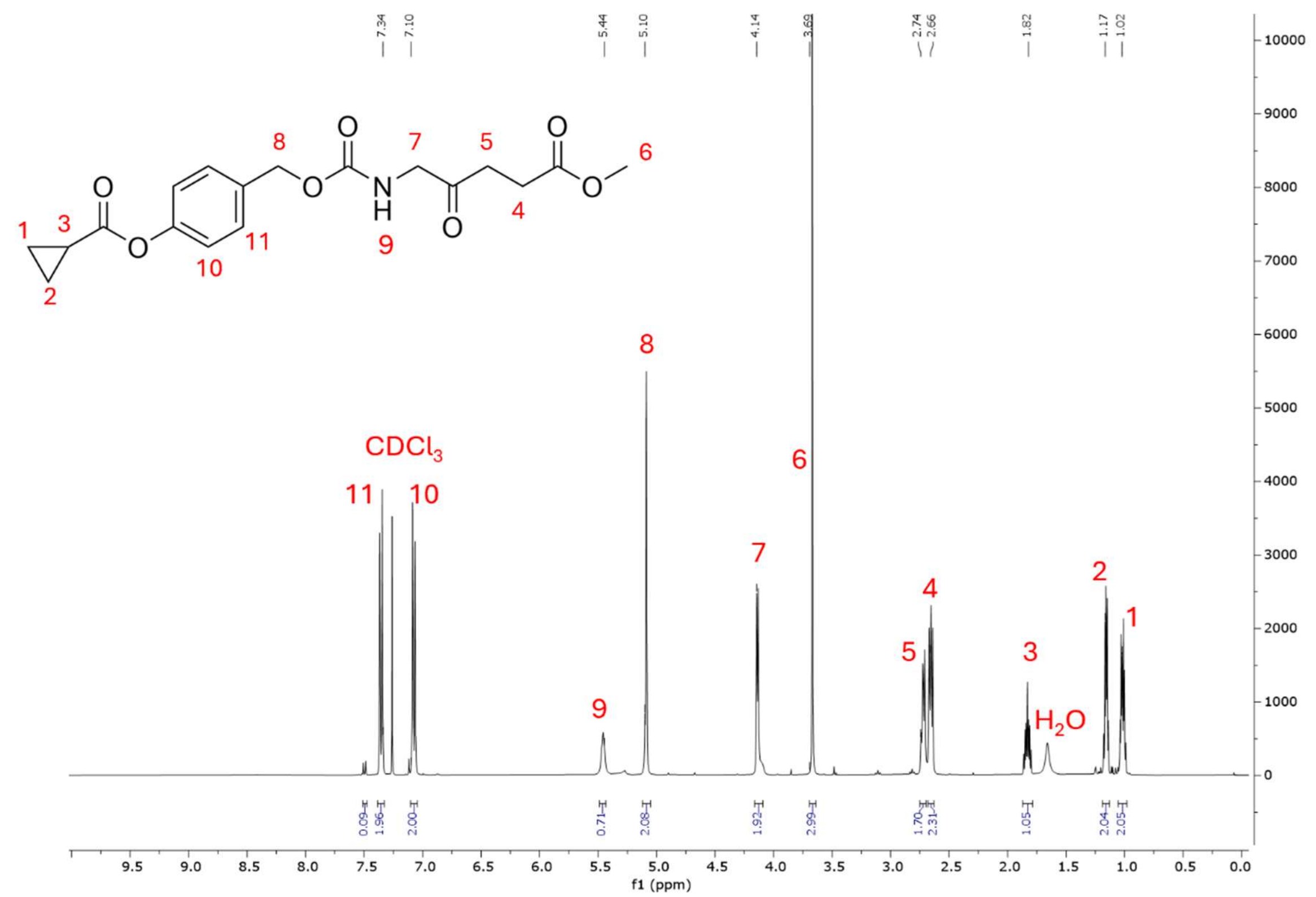

^{1}H NMR (400 MHz, $CDCl_3$) of **5-eMAL**

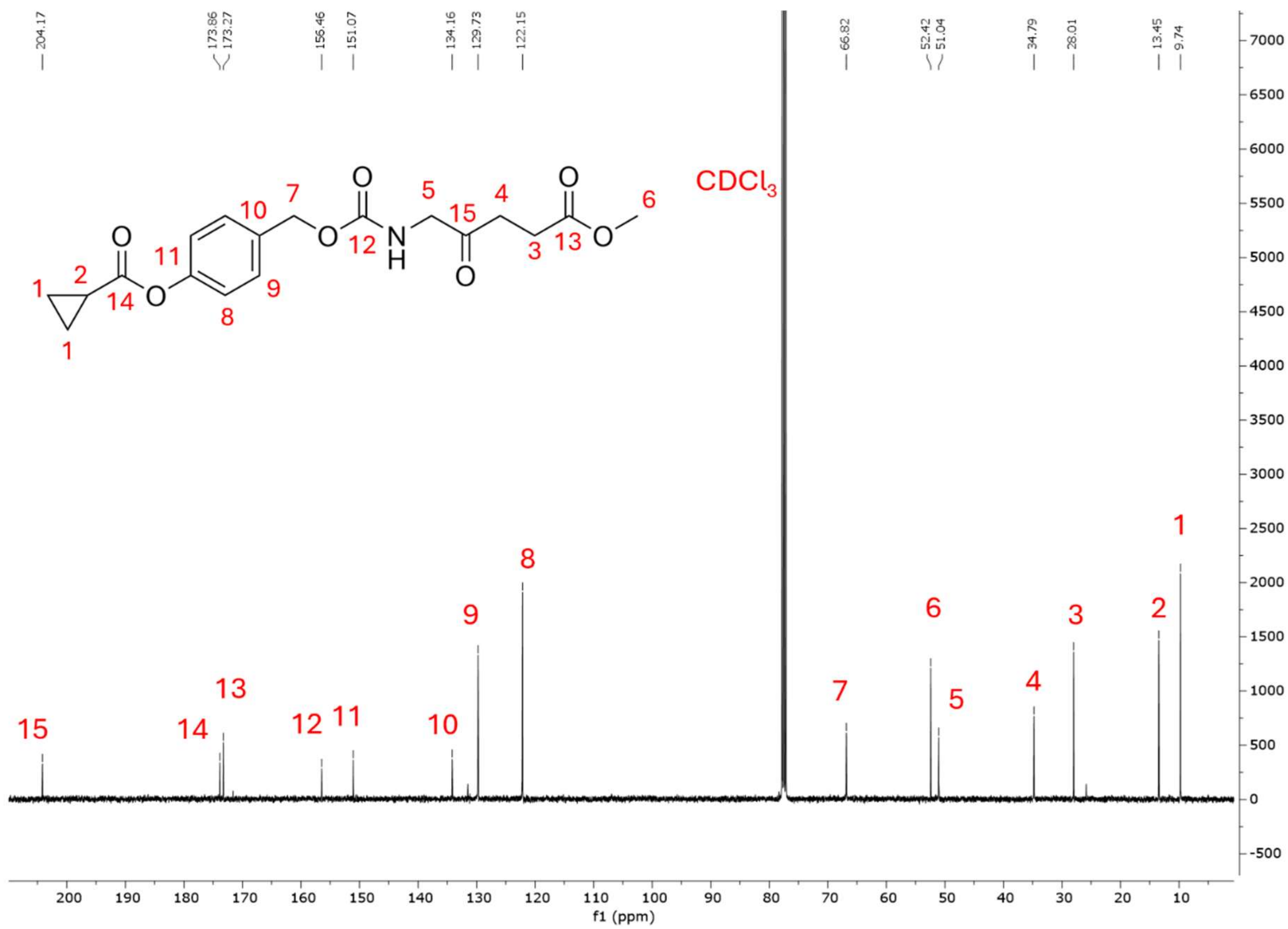

^{13}C NMR (400 MHz, $CDCl_3$) of **5-eMAL**

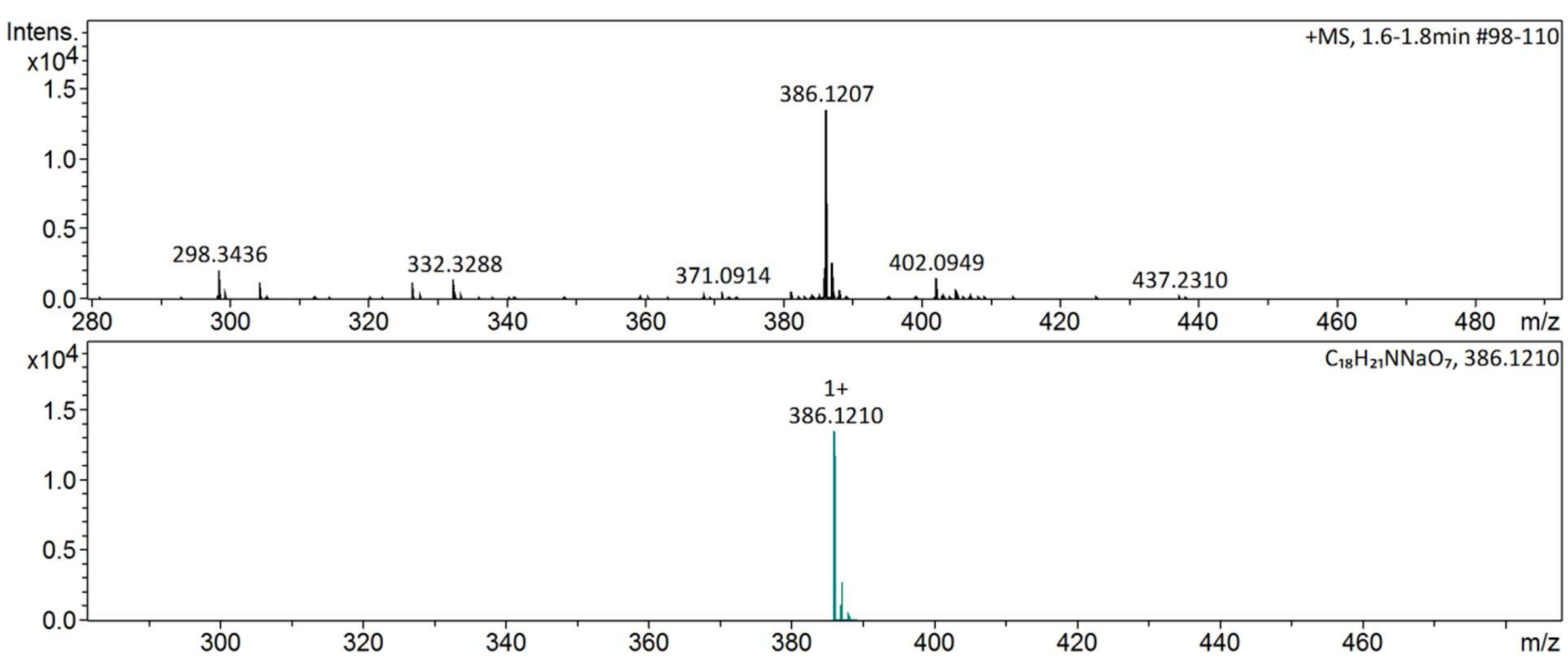

HRMS (ESI-TOF) spectrum of **5-eMAL**

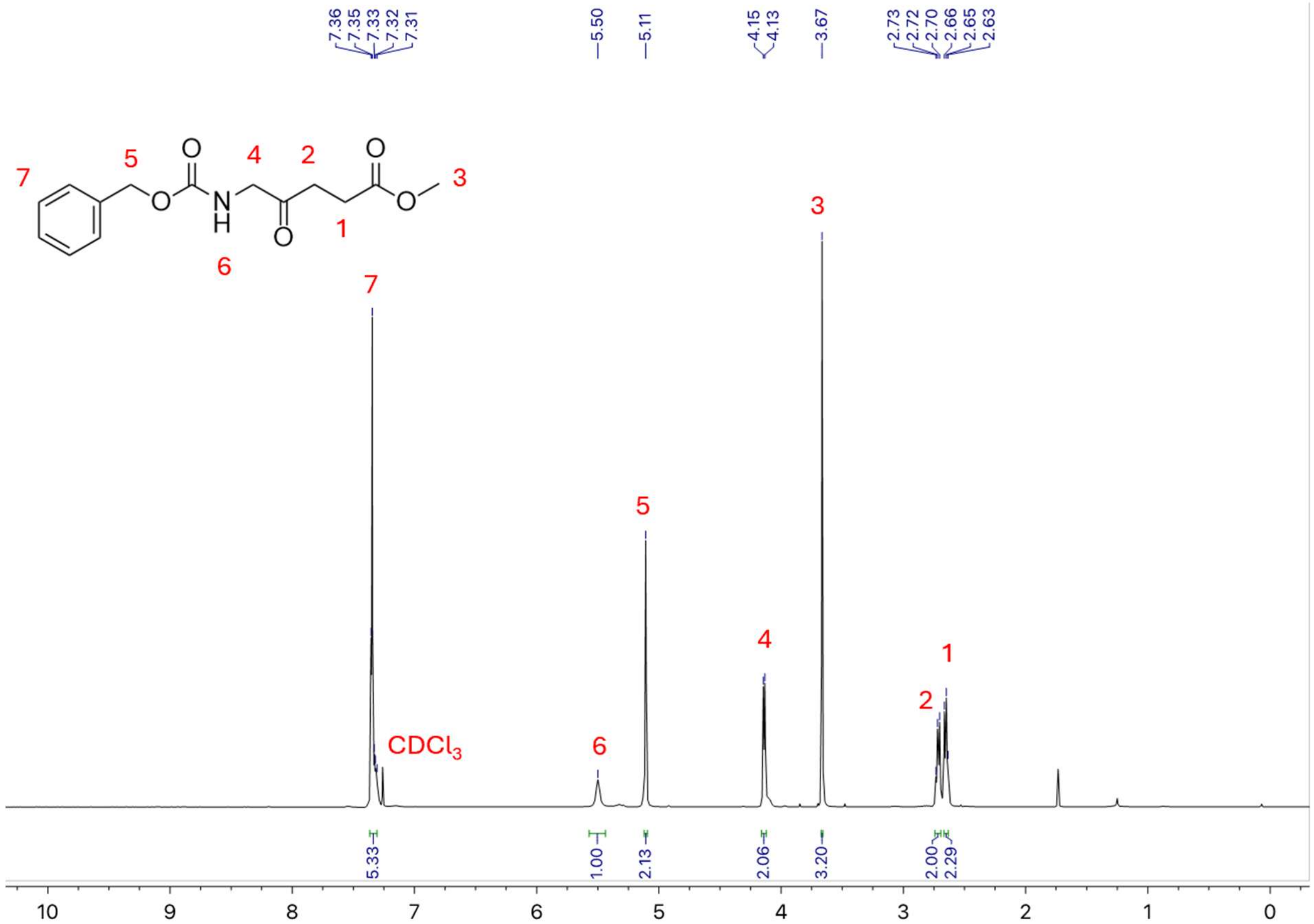

^{1}H NMR spectrum (400 MHz, $CDCl_3$) of **5-bMAL**

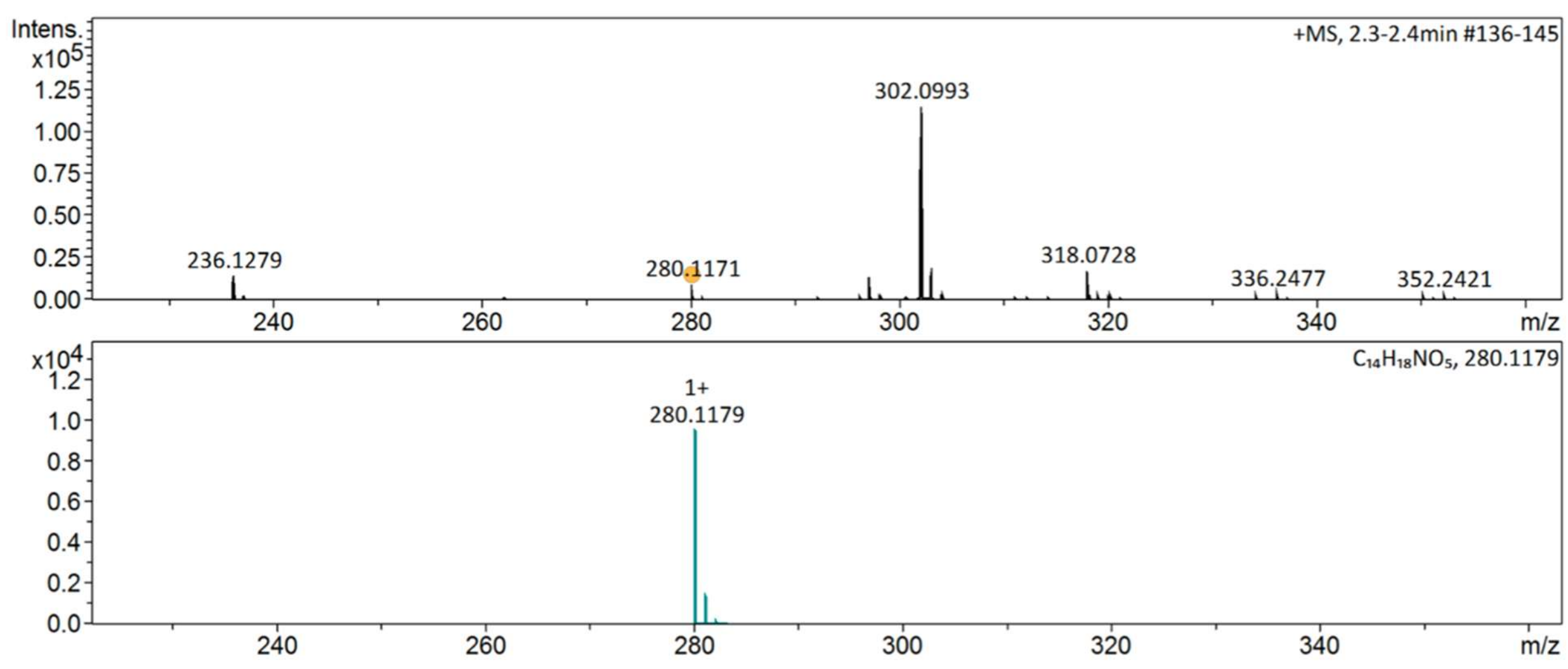

HRMS (ESI-TOF) spectrum of **5-bMAL**

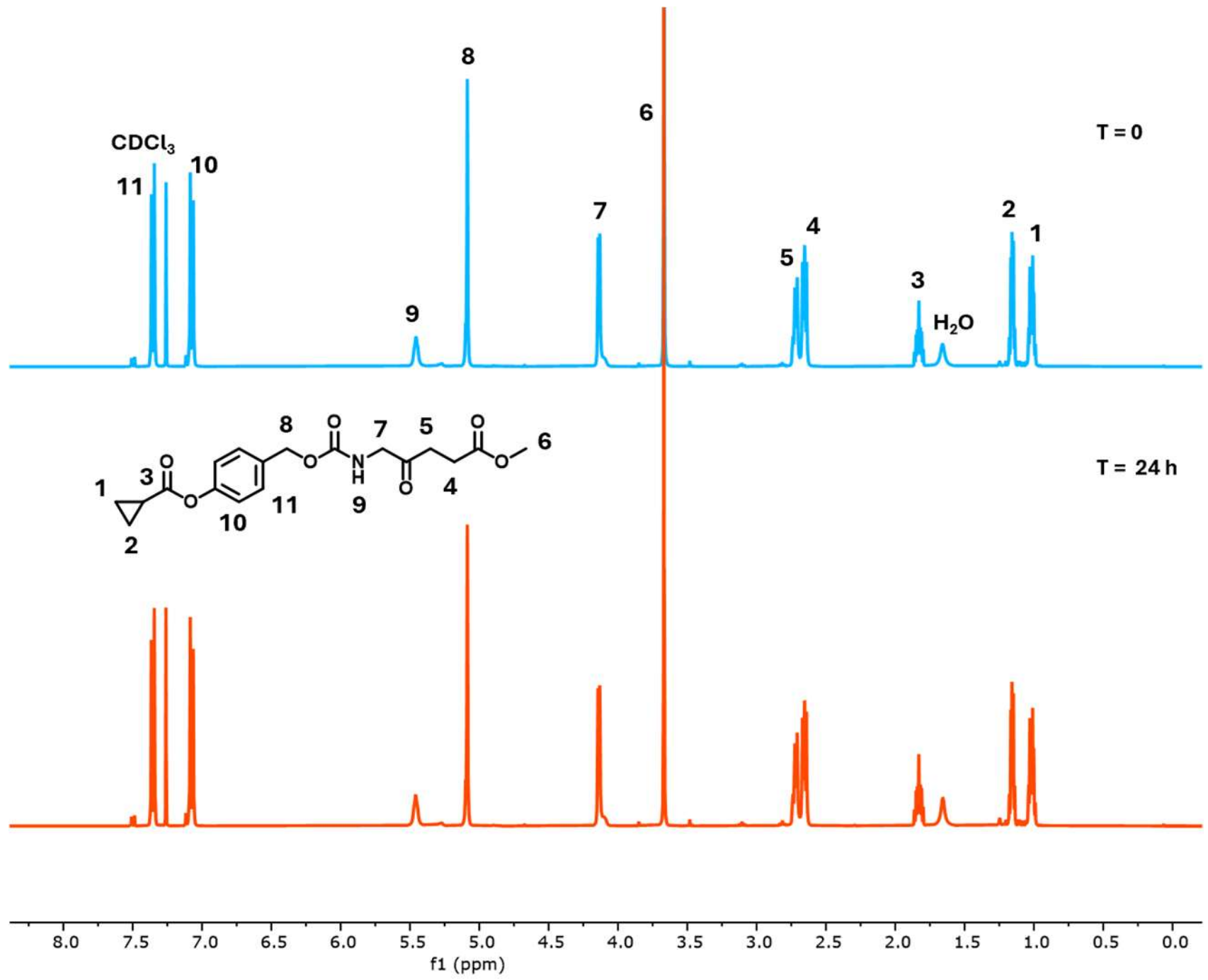

Scheme S1: ^{1}HNMR spectrum (400 MHz, $CDCl_3$, 25°C) showing that a solution of 5-eMAL is stable over 24 hours at room temperature.

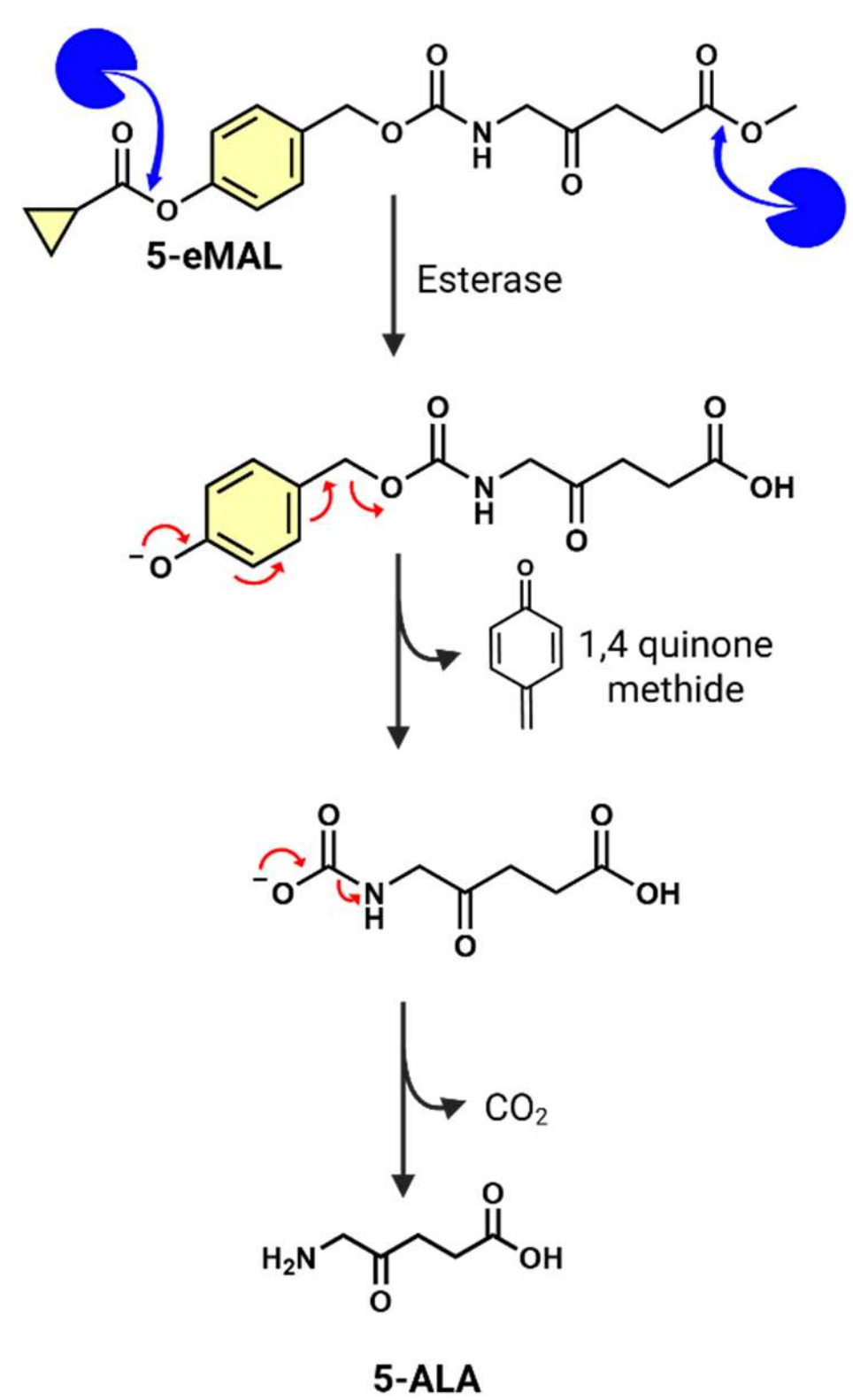

Scheme S2: Esterase triggered conversion of 5-eMAL into 5-ALA.

Biological Controls

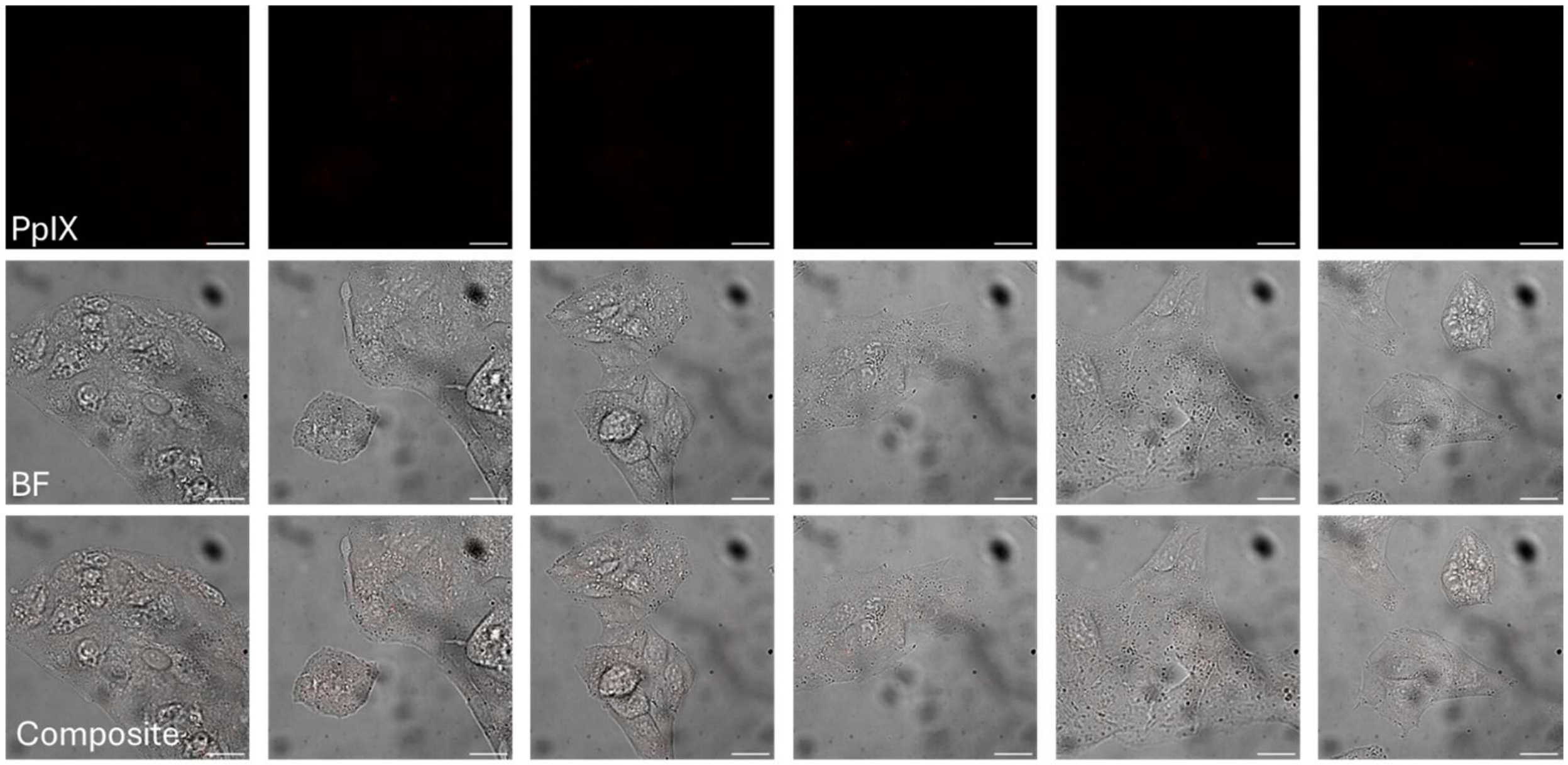

Figure S1: Micrographs of untreated HepG2 cells. Ex: 387/11 nm; Dichromatic mirror: 409 nm; Em: 655/40 nm. Scale Bar = 30 µm.

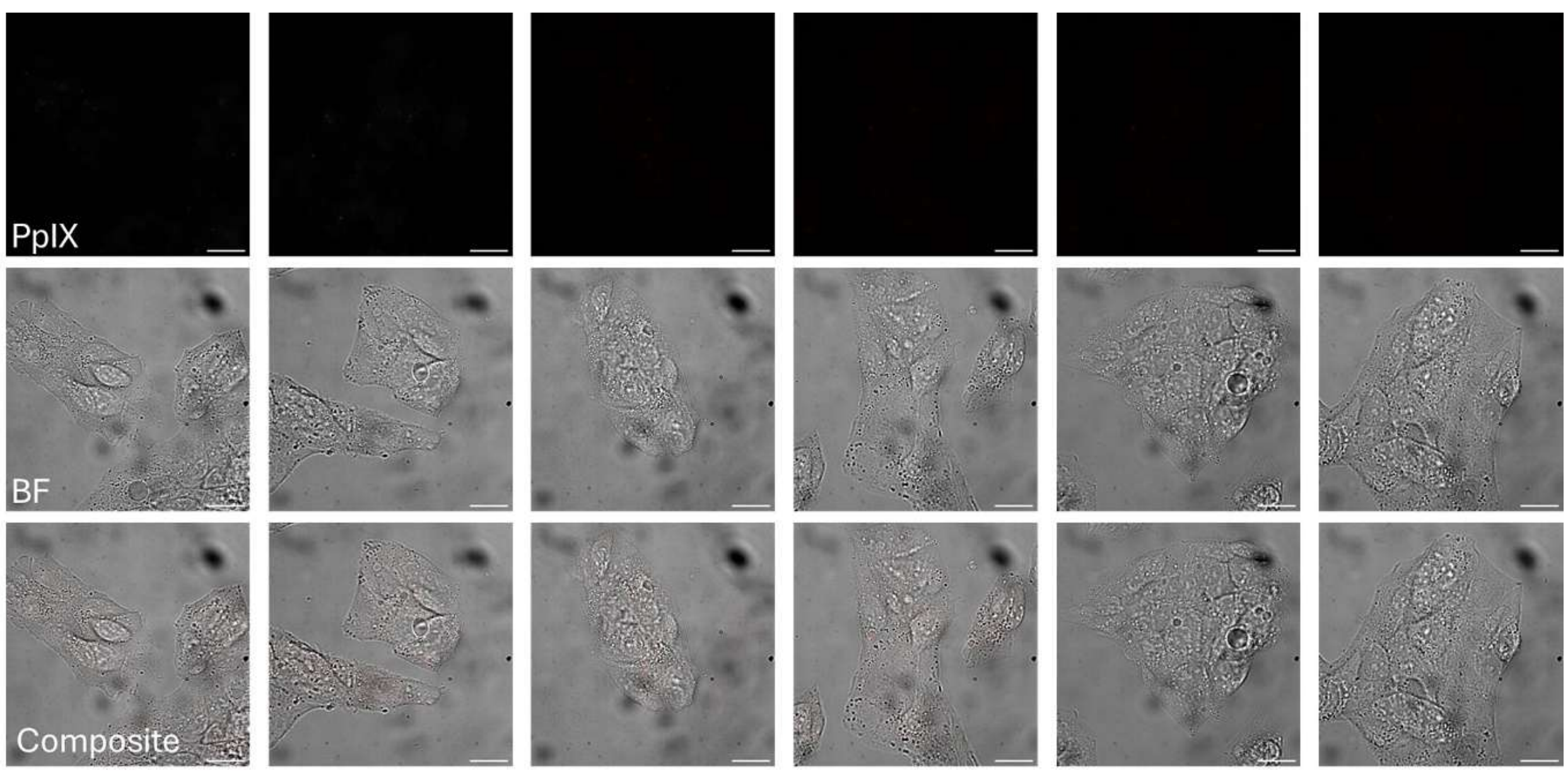

Figure S2: Micrographs of HepG2 cells incubated three hours with 5-ALA (5 µM). Ex: 387/11 nm; Dichromatic mirror: 409 nm; Em: 655/40 nm. Scale Bar = 30 µm.

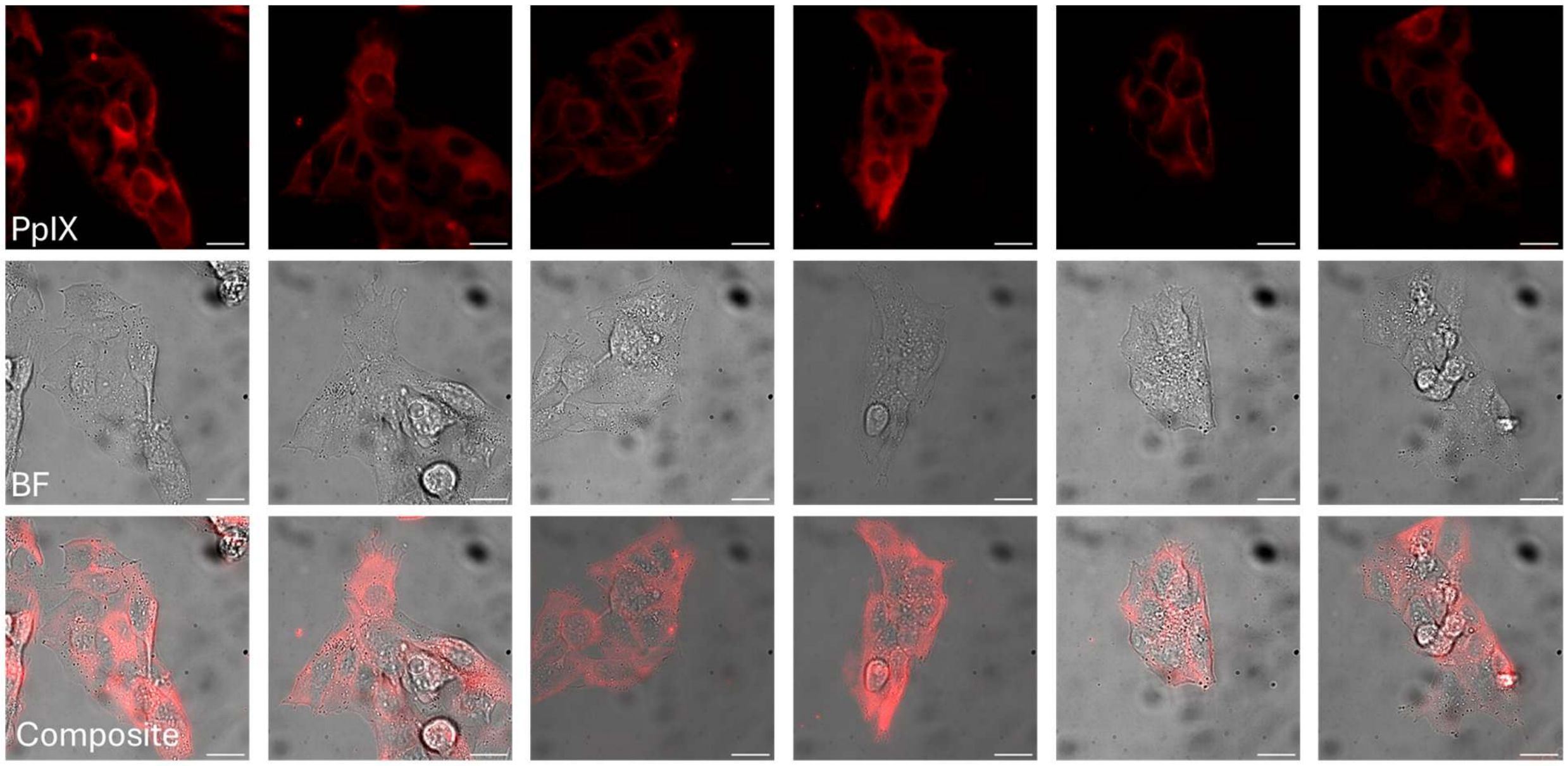

Figure S3: Micrographs of HepG2 cells incubated three hours with 5-eMAL (5 µM). Ex: 387/11 nm; Dichromatic mirror: 409 nm; Em: 655/40 nm. Scale Bar = 30 µm.

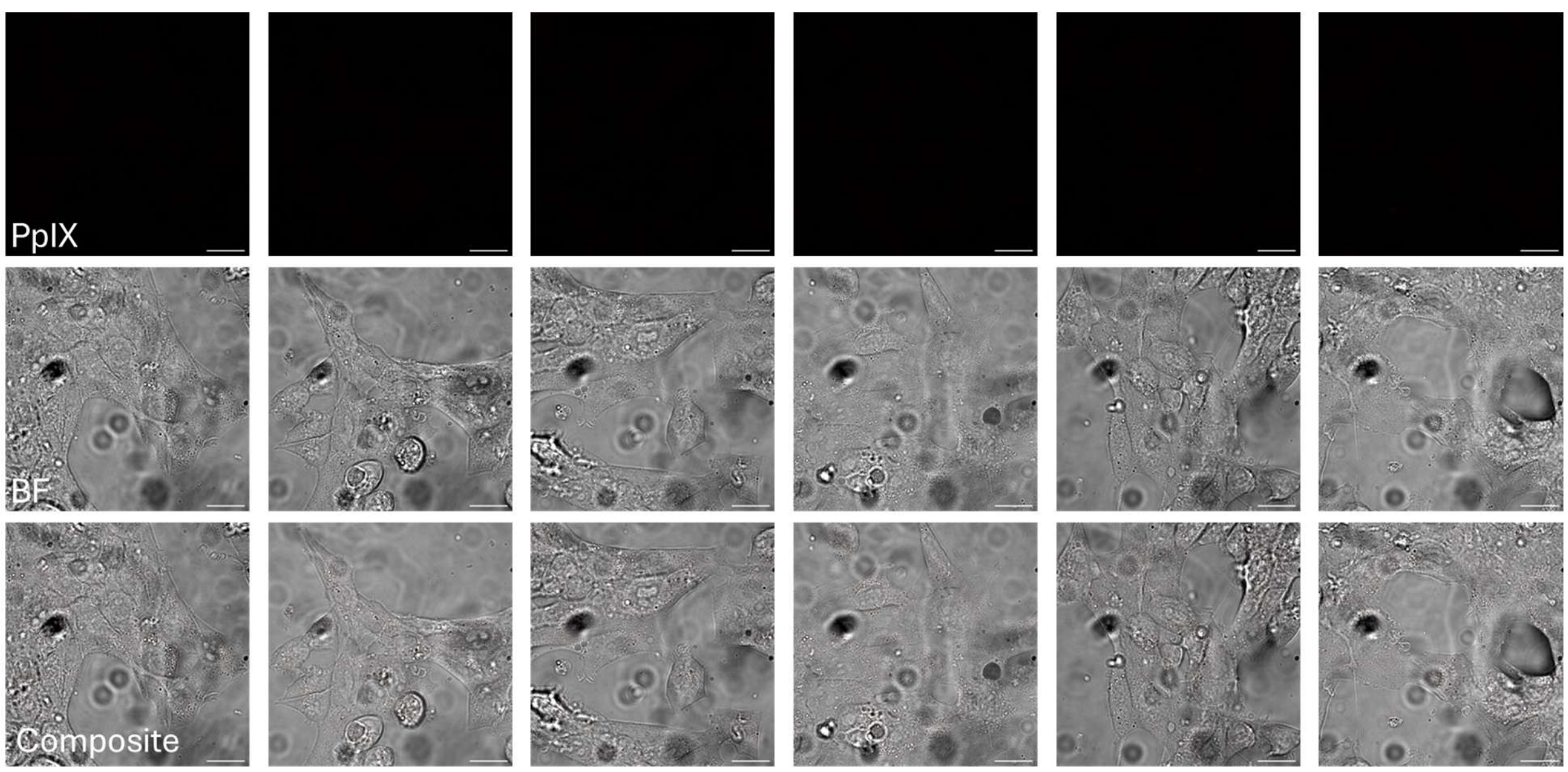

Figure S4: Micrographs of untreated 4T1 cells. Ex: 387/11 nm; Dichromatic mirror: 409 nm; Em: 655/40 nm. Scale Bar = 30 µm.

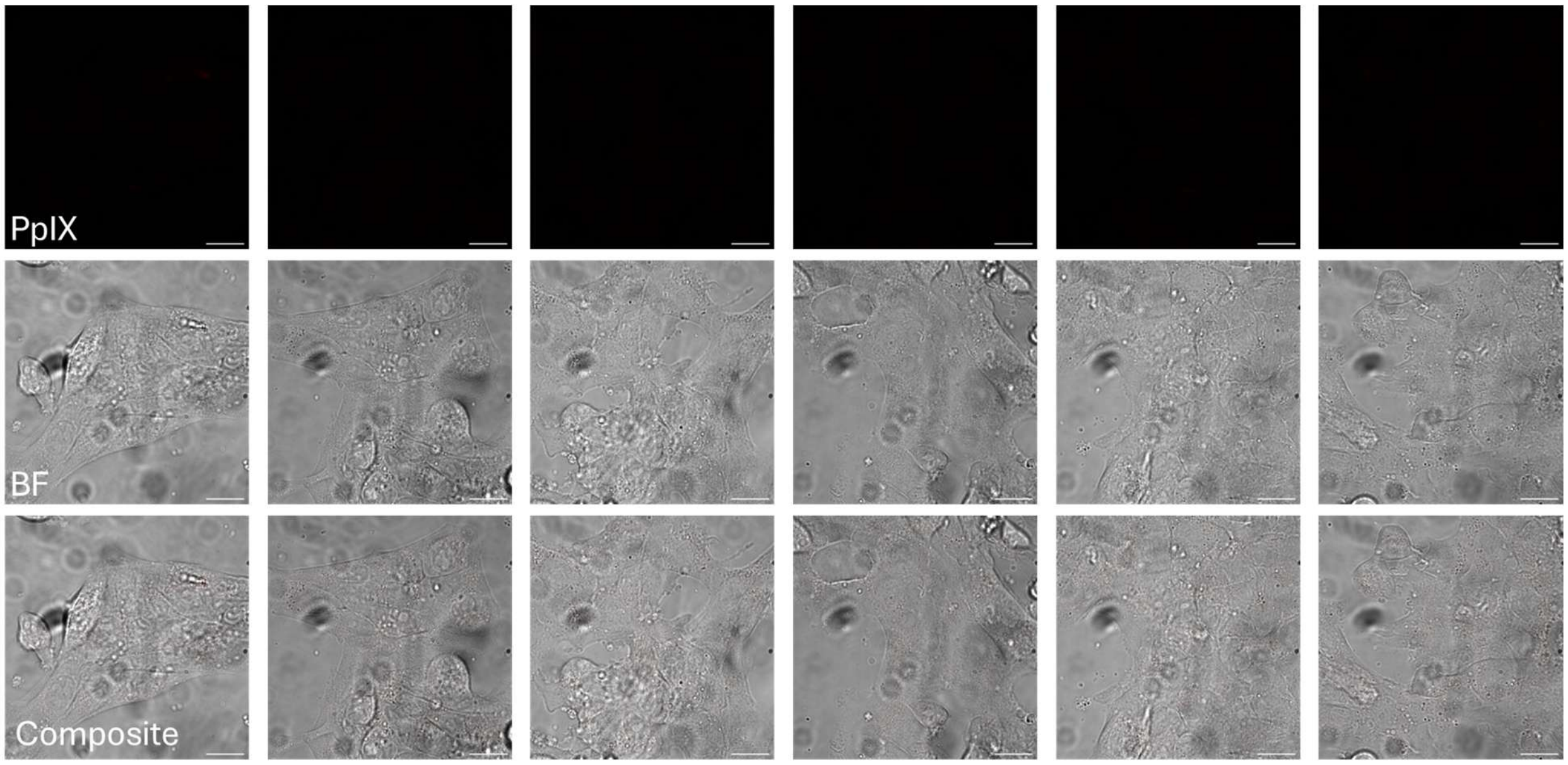

Figure S5: Micrographs of 4T1 cells incubated three hours with 5-ALA (5 µM). Ex: 387/11 nm; Dichromatic mirror: 409 nm; Em: 655/40 nm. Scale Bar = 30 µm.

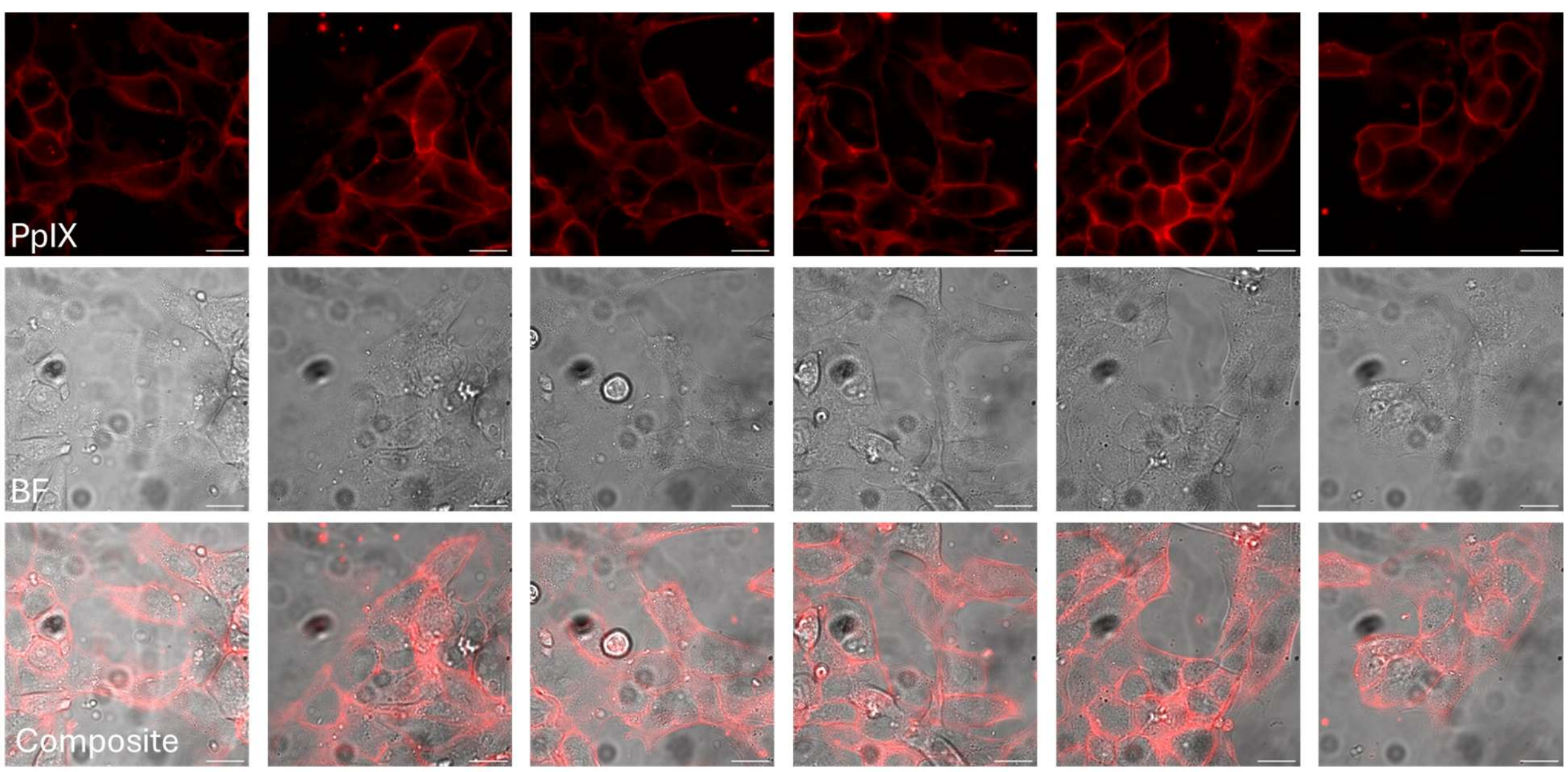

Figure S6: Micrographs of 4T1 cells incubated three hours with 5-eMAL (5 µM). Ex: 387/11 nm; Dichromatic mirror: 409 nm; Em: 655/40 nm. Scale Bar = 30 µm.

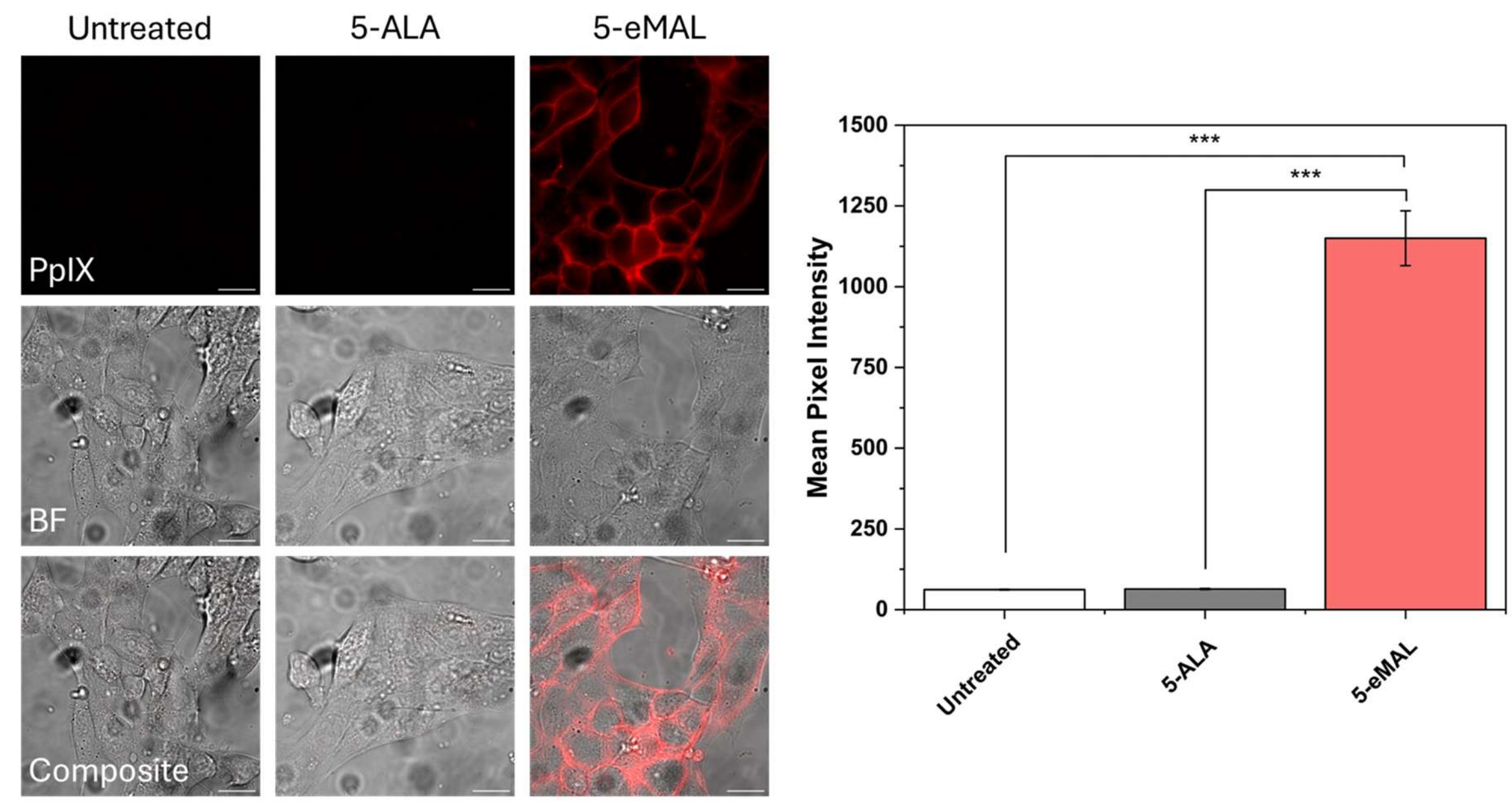

Figure S7: (a) Representative micrographs of 4T1 cells left untreated, incubated three hours with 5-ALA (5 µM) or 5-eMAL (5 µM). (b) Average mean pixel intensity of 4T1 cells. Bars represent standard error (N = 6). *** $p<0.001$. Ex: 387/11 nm; Dichromatic mirror: 409 nm; Em: 655/40 nm. Scale bar = 30 µm.

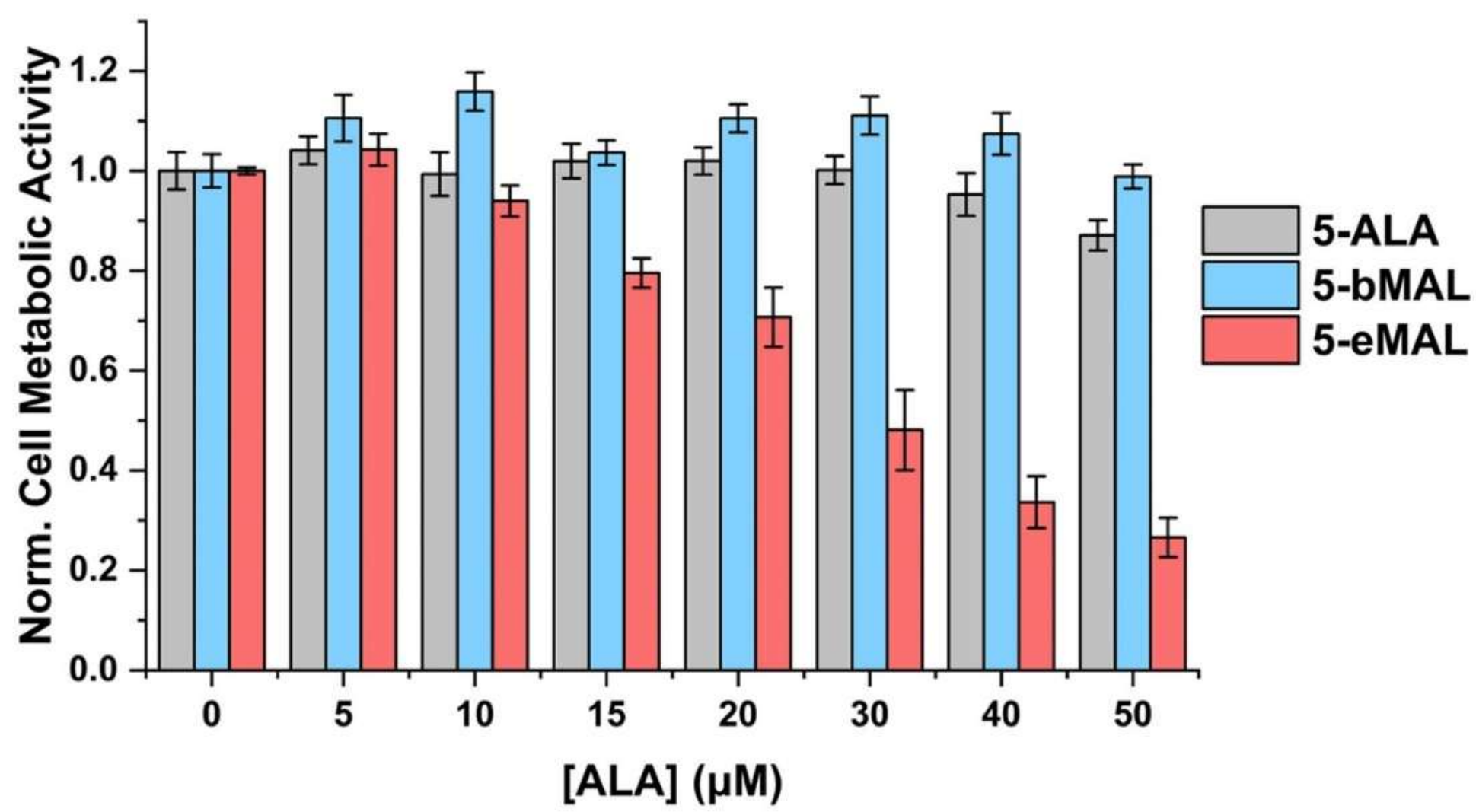

Figure S8: Normalized metabolic activity of HepG2 cells incubated for 24 hours with either 5-ALA, 5-eMAL, or 5-bMAL (0 – 50 µM) in dark. Bars represent standard error of one experiment where N = 3 replicates of the same concentration.

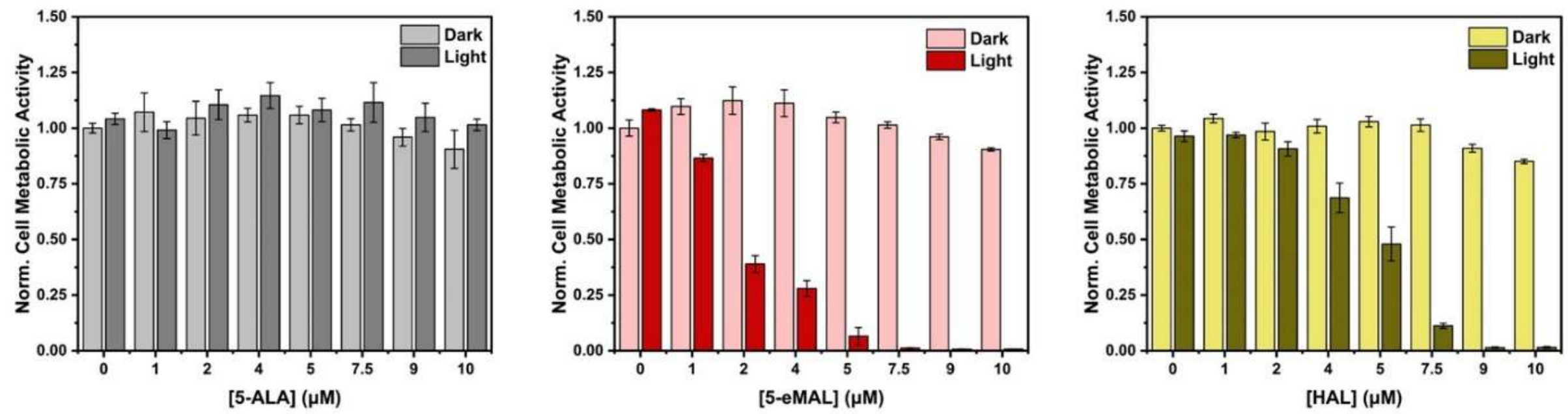

Figure S9: Normalized metabolic activity of HepG2 cells incubated for 3 hours with 5-ALA, 5-eMAL, or HAL (0 – 10 μM) and kept in dark or cells incubated 3 hours with 5-ALA, 5-eMAL, or HAL (0 – 10 μM) and then irradiated with 405 nm light (6 mW) for 10 minutes. Bars represent error where N = 3.

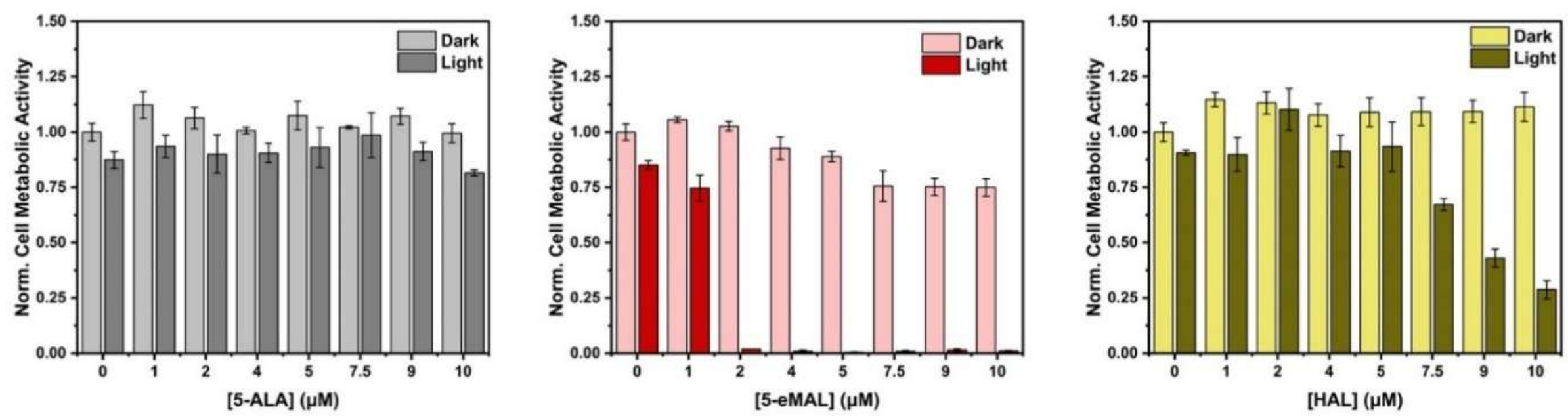

Figure S10: Normalized metabolic activity of 4T1 cells incubated for 3 hours with 5-ALA, 5-eMAL, or HAL (0 – 10 μM) and kept in dark or cells incubated 3 hours with 5-ALA, 5-eMAL, or HAL (0 – 10 μM) and then irradiated with 405 nm light (6 mW) for 10 minutes. Bars represent error where N = 3.
